# Extreme home range sizes among Eurasian lynx at the northern edge of their biogeographic range

**DOI:** 10.1002/ece3.7436

**Published:** 2021-03-18

**Authors:** John D.C. Linnell, Jenny Mattisson, John Odden

**Affiliations:** ^1^ Norwegian Institute for Nature Research Trondheim Norway; ^2^ Norwegian Institute for Nature Research Oslo Norway

**Keywords:** carnivore, habitat, home range, ranging behavior, scandinavia

## Abstract

Eurasian lynx (*Lynx lynx*) have a wide distribution across Eurasia. The northern edge of this distribution is in Norway, where they reach up to 72 degrees north. We conducted a study of lynx space use in this region from 2007 to 2013 using GPS telemetry. The home range sizes averaged 2,606 (± 438 SE) km^2^ for males (*n* = 9 ranges) and 1,456 (± 179 SE) km^2^ for females (*n* = 24 ranges). These are the largest home ranges reported for any large felid, and indeed are only matched by polar bears, arctic living wolves, and grizzly bears among all the Carnivora. The habitat occupied was almost entirely treeless alpine tundra, with home ranges only containing from 20% to 25% of forest. These data have clear implications for the spatial planning of lynx management in the far north as the current management zones are located in unsuitable habitats and are not large enough to encompass individual lynx movements.

## Graphical abstract

The northern edge of the Eurasian lynx (*Lynx lynx*) distribution is in Norway, where they reach up to 72 degrees north. Here, we observed the largest home ranges reported for any felid, averaged 2,606 (± 438 SE) km^2^ for males and 1,456 (± 179 SE) km^2^ for females. The habitat occupied was almost entirely treeless alpine tundra, with home ranges only containing from 20 to 25 % of forest.

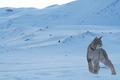

## 1 INTRODUCTION

Knowledge concerning the spatial requirements of wildlife is one of the most basic requirements in conservation planning in an increasingly human‐dominated world. This concerns both the extent of space use and the range of habitat tolerances and preferences for any given species. This knowledge is essential for issues such as choosing the location and size of protected areas and management units (Linnell et al., 2001; Woodroffe & Ginsberg, 2000) such that they are large enough to fully embrace the home ranges of multiple individuals and are located in areas that provide suitable habitat. Furthermore, movement data is essential for the design and interpretation of monitoring data (Linnell, 2015; Linnell et al., 2001, 2005, 2007; Rowcliffe et al., 2008).

Although fundamental, such data are often very expensive and logistically challenging to collect because it involves animal capture and the use of radio‐ or GPS‐telemetry, at least for wide‐roaming cryptic animals like large carnivores. Clearly it is not possible to conduct such studies in all parts of all species distributions. Accordingly, a central question concerns the extent to which research results can be transferred between sites, and extrapolated from single sites to wider areas of a species distribution (Fahey et al., 2015). Multiple studies have explored the factors affecting home range size variation in mammals, especially carnivores, at both the intra‐ and interspecific levels. These studies have identified a set of factors that influence home range size. These include both intrinsic properties such as sex, body weight, and feeding style, and extrinsic factors like population density, prey density, and patterns of environmental productivity (Duncan et al., 2015; Kelt & Van Vuren, 2001; Nilsen et al., 2005). As a result, it is possible to predict a great deal of the within and between study site variation in species’ space use patterns (e.g., Jedrzejewski et al., 2007; Mattisson et al., 2013; Kittle et al., 2015 for wolves, *Canis lupus*). However, there is still much unexplained variation for generalist species with wide distributions, where different regions may differ dramatically in terms of the prey species that are available and habitat structure (e.g., Walton et al., 2001).

The Eurasian lynx (*Lynx lynx*) is such a species, distributed across the Palearctic from western Europe to the Pacific coast of Siberia, and from the northern boreal forests to the Central Asian grasslands and Himalayan mountains (Breitenmoser et al., 2015). Despite this very large range, the vast majority of telemetry studies have been conducted in central Europe (Switzerland, Germany, Poland) and southern Scandinavia, in heavily forested sites, and where roe deer (*Capreolus capreolus*) are available and constitute the main prey (e.g., Andrén & Liberg, 2015; Breitenmoser et al., 1993; Breitenmoser‐Würsten et al., 2007; Linnell et al., 2001; Schmidt et al., 1997; Stahl et al., 2002). Between these sites the variation in home range size is related to roe deer density and environmental productivity (Herfindal et al., 2005) and/or lynx density (Aronsson et al., 2016; Pesenti & Zimmermann, 2013), although their home range sizes are consistently larger than would be predicted from their body size alone (Duncan et al., 2015). A major question concerns the extent to which these ecological insights can predict their behavior in areas without roe deer, and where forest is scarce.

**FIGURE 1:**
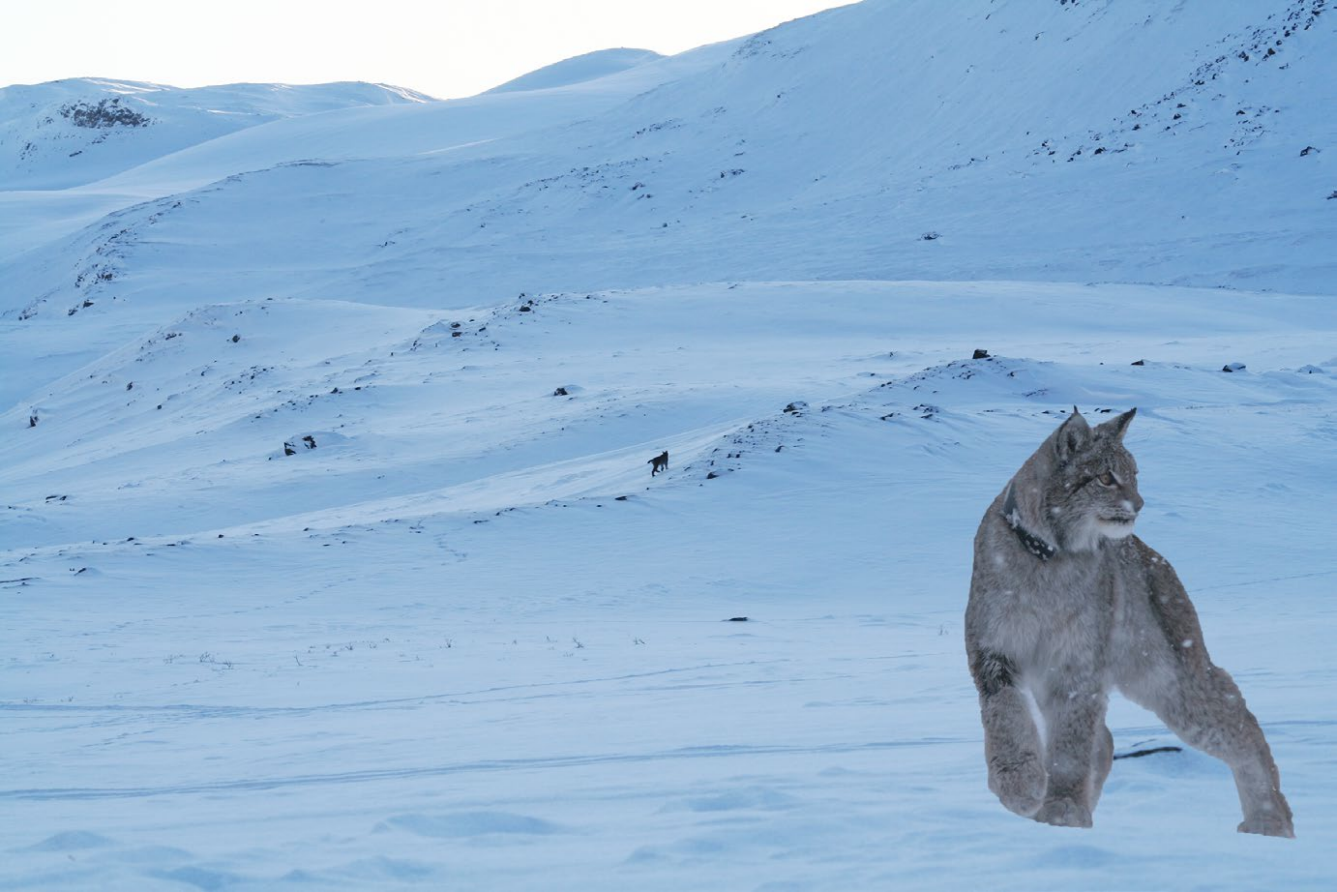
A Eurasian lynx *Lynx lynx* (small object in the middle of the photo) in the tundra of Northern Norway (photo: John Odden). Inserted photo shows a close up of one of the GPS‐collared female lynx in winter coat (photo: John Ivar Larsen)

This paper presents new data on the spatial ecology of the Eurasian lynx, at the extreme northern edge of its distribution along the Barents Sea coast of northern Norway, where the landscape is dominated by alpine tundra (Figure 1), and semi‐domestic reindeer (*Rangifer tarandus*) constitute the only abundant large herbivore prey (roe deer are not present in significant numbers, Mattisson, Odden, et al., 2011). Historically, lynx were absent from this region of Norway before the late 1980's, but have been consistently present since then (Linnell et al., 2010). Our research objective was to quantify lynx space use and home range composition in this specific ecosystem which differs greatly from almost all other sites where lynx have been studied. As previous reviews have found a negative relationship between environmental productivity and home range size (Duncan et al., 2015; Herfindal et al., 2005), and in line with literature on peripheral populations (Koprowski et al., 2008), we expected that home ranges would be exceptionally large in this site. The resulting estimates of home range size, as well as data on the presence of lynx throughout the region, are then related to the size and location of different wildlife management zones.

## 2 MATERIALS AND METHODS

We captured and fitted lynx with GPS‐collars, estimated home ranges from the GPS locations, classified broad scale habitat within the home ranges, and evaluated the appropriateness of lynx management zones using our home ranges estimates. We also determined the distribution and density of lynx throughout the study area using location data from public reports. We conducted field work from 2007 to 2013. All lynx were darted from helicopters and immobilized with medetomidine‐ketamine (Arnemo et al., 2012). We equipped animals with GPS collars with GSM download (Vectronic Aerospace Gmbh, Berlin, Germany; Televilt, Lindesberg, Sweden). The handling protocols were approved by both the Norwegian animal research ethics committee and wildlife management authority. Location frequencies varied over time, with a basal frequency of two locations per day, interspersed with intensive periods of up to hourly locations when predation studies were being conducted (Mattisson, Arntsen, et al., 2014; Mattisson, Odden, et al., ,^2011^, ^2014^). We recaptured and re‐collared several animals to secure more than one year's data (battery life of the collar was normally < 12 months). In this study, we only include data on adult individuals that clearly occupied stable home ranges (i.e., were not transient or dispersers).

**FIGURE 3:**
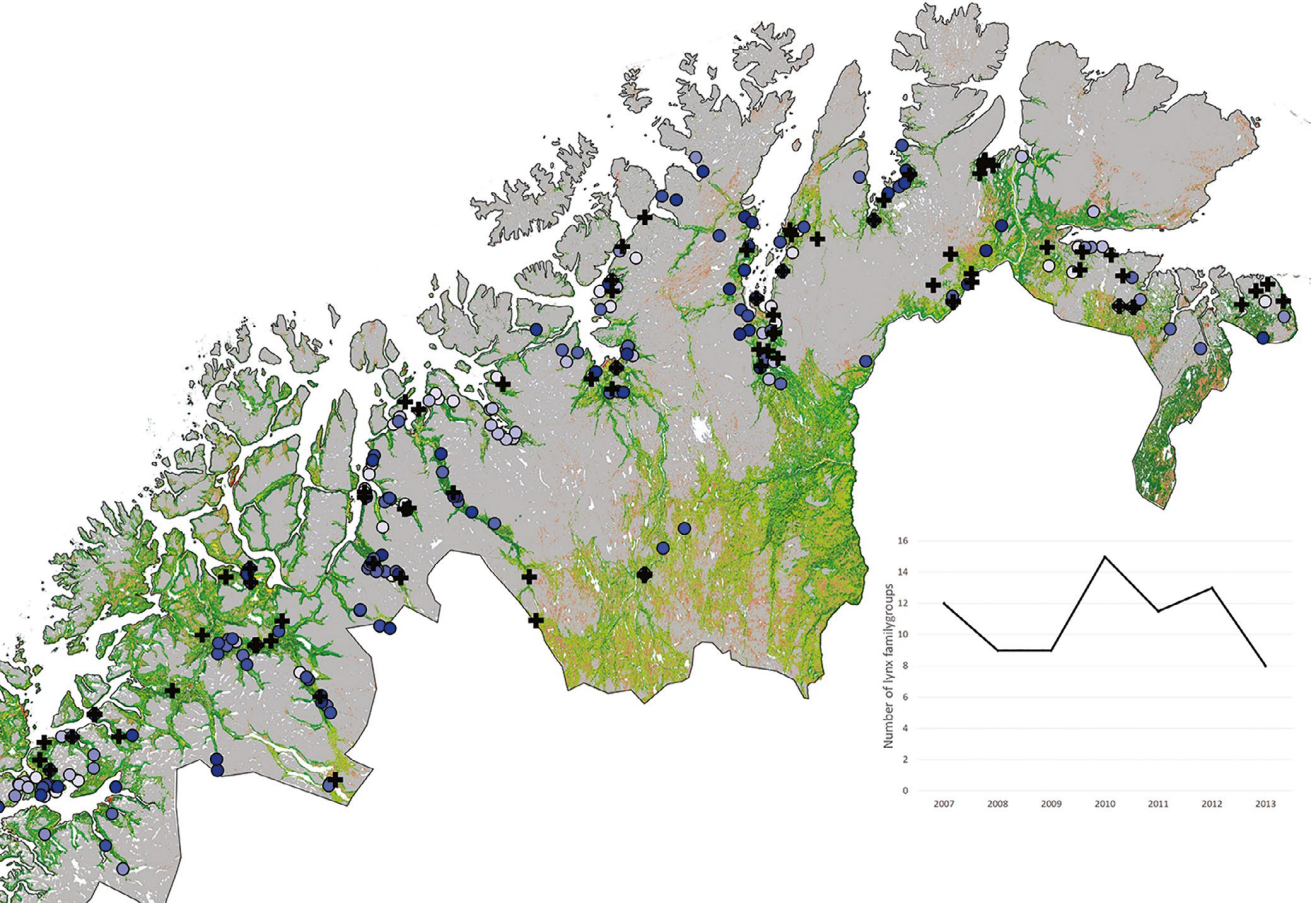
Map of lynx (*Lynx lynx*) distribution records in Troms and Finnmark during 2007–2013 based on lynx shot or found dead (crosses), and recorded tracks of family groups during winter (blue spots where color strength represents different years, lightest in 2007 and darkest in 2013). Gray areas are open tundra while green represent forested areas. The graph shows the annual number of family groups detected in the region

Prior to estimating home ranges, we visually screened GPS locations for extreme outliers resulting in the removal of 0.07% of the 56,102 available locations. Because the study focuses on the area occupied, rather than identifying intensity of use of different areas, and because the location frequency was high we did not use probabilistic home range estimators. Instead, we represent home ranges using three polygon methods, the 100% Minimum Convex Polygon, the 95% Minimum Convex Polygon and Concave Polygons with restricted edge set to 0.5 (using software Ranges 8). The results are presented as the 100% MCP unless otherwise indicated. Because our focus was on annual home ranges, we only present data on individuals with at least 10 months of location data for a given year. All areas falling into the sea were removed from the home range estimates, as these are not used by the lynx. In order to maximize the number of individuals and align analysis to the timing of capture (usually February) we defined the year as extending from February 1st to January 31st. We estimated spatial overlap between ranges of MCP 100% as ({Area of overlap A‐B/Area A} + {Area of overlap A‐B/Area B})/2 where A and B represent either different individuals within the same year or the same individual in different year. We extracted the proportion of forest in home ranges from a national vegetation map (30x30m raster; using main class forest vegetation—finer class 1–8 in Johansen et al. 2009; Figure 3).

We obtained data on the distribution of lynx in the study area from the National Large Predator Monitoring Program (www.rovdata.no). During winter, the public are encouraged to report observations of tracks in the snow from multiple lynx, which generally constitute females with dependent kittens. All such reports are located and examined by trained personnel, and are used to come up with an index of population size (number of family groups) for the region (Andrén et al., 2002; Gervasi et al., 2013; Linnell et al., 2007). In addition, locations of all lynx killed in the annual quota‐regulated harvest (Linnell et al., 2010), shot in damage‐control operations, killed in traffic accidents or otherwise found dead are collected by the wildlife management agencies and carcasses of the dead lynx are sent in for autopsy at the authors’ institution (www.rovdata.no).

In order to evaluate the appropriate size and location of the management zones, we visually overlaid the telemetry data with the borders of the management zones (Miljødirektoratet—Kartkatalog (miljodirektoratet.no)) and major protected areas (http://kartkatalog.miljodirektoratet.no).

### 2.1 Study site

**FIGURE 2:**
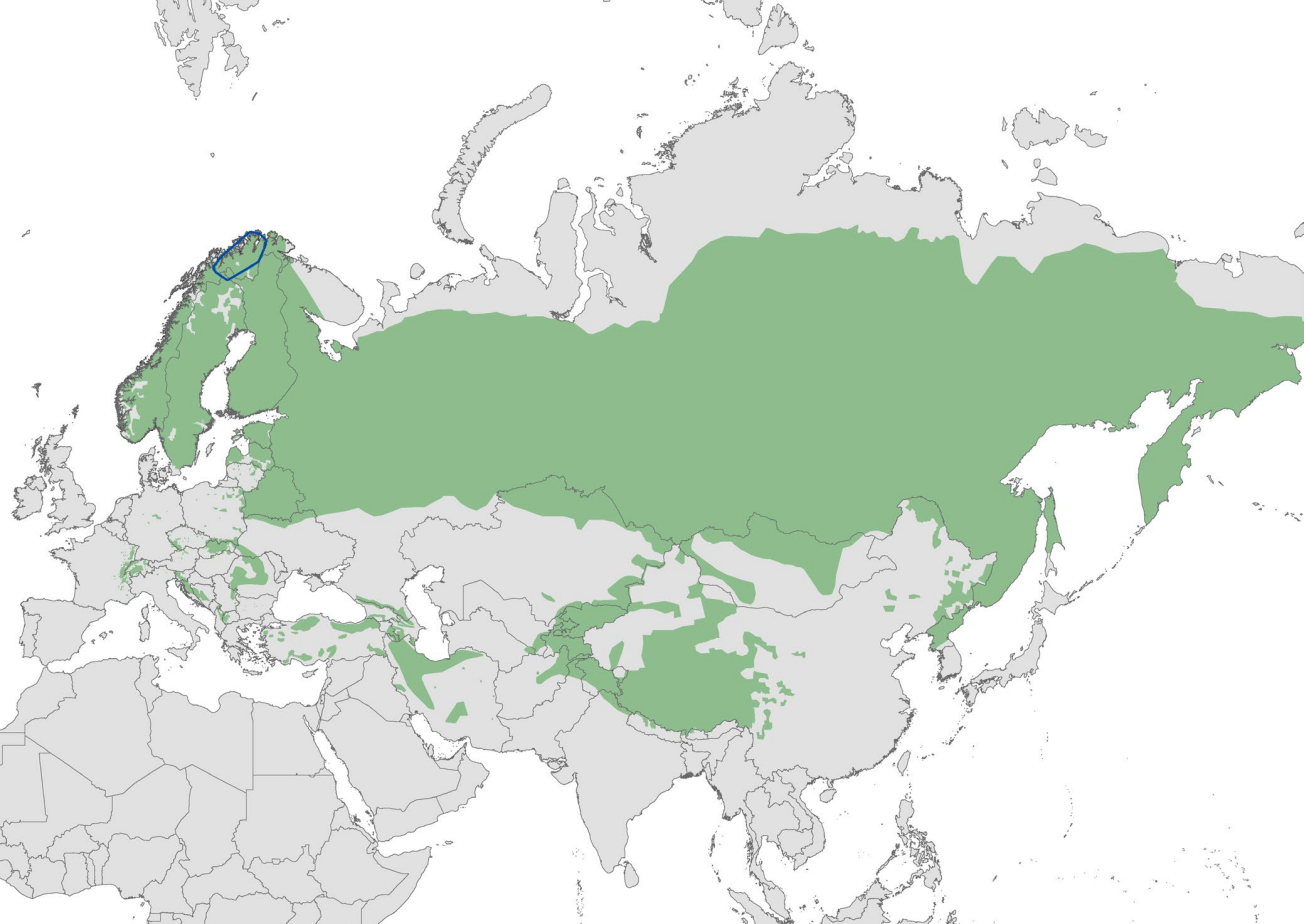
Distribution of Eurasian Lynx *Lynx lynx* (green area) where the blue polygon represent the area in northern Norway where the telemetry study was conducted. The distribution map was downloaded from the International Union for the Conservation of Nature, The IUCN Red List of Threatened Species at https://www.iucnredlist.org (20 January 2021)

We conducted this study in the counties of Troms and Finnmark in northern Norway (69° to 71° N, 20° to 25° E; Figure 2). This is the northernmost part of the species distribution where the European continent meets the Barents Sea. There is strong seasonality in both climate and light conditions. During winter, there are 54 polar nights (24 hr darkness) and 72 polar days during summer (24 hr light) (Heurich et al., 2014). The climate is strongly influenced by the coast, which remains mainly ice‐free during winter. This gives a milder climate than expected for the extreme latitude, although the effect becomes weaker with increasing distance from the coast. The overall climate is coastal‐alpine along the coast, and continental in the inland areas. Snow usually lies on the ground from November to April, although some precipitation can fall as rain in any month. Alpine tundra vegetation dominates the study area, with mountain birch (*Betula pubescens*) forest occurring along the coast and low lying river valleys. Some few patches of pine (*Pinus sylvestris*) forest also occur in valley bottoms. Human density is low (3.2 per km^2^) and there is little human infrastructure or habitat modification. The topography is complex, with many convoluted fjords at the coast which is fringed by mountains rising to altitudes of 500 to 1,400 m. These mountains are punctuated with steep glacial valleys. The inland area consists of a more undulating topography at altitudes of between 400 and 800 m.

The main prey of lynx throughout Europe, the roe deer, is functionally absent from the study area. As a consequence, the only available ungulate prey are semi‐domestic reindeer (*Rangifer tarandus*, Mattisson, Odden, et al., 2011). Semi‐domestic reindeer are free‐ranging year around, but gathered by the herder who own them a few times a year for the marking of calves, slaughtering, and to be actively herded between winter and summer ranges which may be separated by more than 100 km. Between these events herding is not intensive, although there is irregular supervision of the herds and some herders have tested different depredation mitigation measures, like calving inside enclosures, to a very limited extent. In this area, summer reindeer ranges are typically on the coast and winter ranges in the inland areas (Walton et al., 2017).

Mountain hares (*Lepus timidus*), tetraonids (*Lagopus lagopus, Lagopus muta, Tetrao urogallus, Tetrao tetrix*), and red foxes (*Vulpes vulpes*) constitute the main alternative prey (Mattisson, Odden, et al., 2011). Moose (*Alces alces*) are present, but are rarely killed by lynx because of their large size. Free‐ranging domestic sheep (*Ovis aries*) are available during summer in some areas and are occasionally killed by lynx (Mattisson et al., 2014). The only other large predators regularly present are wolverines (*Gulo gulo*) and golden eagles (*Aquila chrysaetos*). Brown bears (*Ursus arctos*) occur at low density to the south and east of our study area, and transient wolves only occasionally disperse through the area.

Lynx are actively managed in the study area through quota regulated hunting and lethal‐control permits with the intention of maintaining the population at a politically determined level in order to limit depredation conflicts on reindeer. During our 7 year study period, the regional lynx population was above the goal for 5 years and below in 2 years. The local management authorities also seek to limit lynx distribution to certain management zones where different regimes apply (Linnell et al., 2010). Within the zones, large carnivores are given higher priority and are in theory allowed to reproduce. Outside the zones livestock are given higher priority and large carnivore numbers are meant to be kept at low density, and are in theory not meant to reproduce. Quota limited hunting is used throughout the region as the main tool to enforce this zoning and to keep the overall population close to the politically set goal.

## 3 RESULTS AND DISCUSSION

During the study period, signs of lynx presence were widely, but thinly, distributed throughout the study area (Figure 3), but with a clear concentration along the coast and the valleys leading in from the coast. Very few lynx were recorded in the interior areas. The number of recorded family groups is an index of population size (Linnell et al., 2007), and annual counts varied between 9 and 15 during the study period (Figure 3). Extrapolating using an assumed population structure (Andrén et al., 2002), the National Monitoring Program estimated the total population size in the two counties (Finnmark and Troms) to have averaged 0.1 lynx per 100 km^2^ during the study period.

GPS‐telemetry data were available for a minimum of 10 months for 4 males (11 annual ranges) and 16 females (24 annual ranges). The observed home ranges, the smallest being 407 km^2^ and the largest 4,805 km^2^ (Table 1), were an order of magnitude larger than those found in other studies conducted on this species in central Europe (Table S1). The only other lynx study that has found ranges even close to these was from the Sarek area of northern Sweden (Mattisson, et al., 2011). Home ranges from this study site were 21% (for males) and 35% (for females) smaller than the ones documented in our study (comparing estimates from concave polygons in both studies). Roe deer are also absent from Sarek, with semi‐domestic reindeer representing the main prey (Mattisson, Odden, et al., 2011; Pedersen et al., 1999).

**TABLE 1:** Annual home range sizes of adult resident Eurasian lynx (*Lynx lynx*) in Troms and Finnmark, northern Norway, 2007–2013, based on three different range estimators. Data are from 4 male lynx (9 annual ranges) and 16 female lynx (24 annual ranges)

	Home range size (km^2^ ± SE)		Proportion of forest in home range	
	Males	Females	Males	Females
MCP (100%)	2,606 (±438)	1,456 (±179)	0.23 (±0.02)	0.20 (±0.01)
MCP (95%)	1857 (±336)	916 (±132)	0.24 (±0.03)	0.26 (±0.02)
Concave	2,243 (±332)	1,195 (±146)	0.24 (±0.02)	0.23 (±0.02)

Individual lynx typically retained the same home range between years (73% overlap ± 2.5; *n* = 13 individuals with 22 ranges). Neighboring female lynx had an average of 22% range overlap (±4.8 SE, *n* = 12 overlapping ranges). We had no neighboring males that were monitored at the same time with sufficient data to calculate overlaps. This degree of overlap between neighboring females is similar to that found in the Sarek area by Mattisson, Persson, et al., (2011), but substantially higher than that documented in Switzerland or Poland (study site averages between 0.2% and 6% overlap; Breitenmoser et al., 1993; Breitenmoser‐Würsten et al., 2007; Schmidt et al., 1997). Although it might be expected that individuals with much larger home ranges maintain less exclusive territories, it is also important to bear in mind that the two Scandinavian studies have used GPS telemetry which collects far more locations (and hence increase chances of detecting overlap) than the older central European studies which were based on VHF telemetry.

These were not only the largest home ranges recorded for the study species (Table S1). They are also the largest home ranges recorded for any wild felid, exceeding the ranges of the much larger Siberian tigers (*Panthera tigris*; average 1,385 km^2^ for males, 390 km^2^ for females) and snow leopards (*Uncia uncia*; average 615 km^2^ for males and 327 km^2^ for females) that also occur in extreme boreal or high altitude environments (Goodrich et al., 2010; Hernandez‐Blanco et al., 2015; Johansson et al., 2016; McCarthy et al., 2010). The only felid ranges that approach our estimates are from cheetahs (*Acinonyx jubatus*) living in desert, or semi‐desert, environments (average 1583 km^2^, Belbachir et al., 2015; one male coalition used 4,862 km^2^, Farhadinia et al., 2013; average 1651 km^2^,Marker et al., 2008). In fact, we could only find two other studies of terrestrial carnivores where adult individuals showed larger average home range sizes. These were from grizzly bears (average 7,245 km^2^ for males, 2,100 km^2^ for females, McLoughlin et al., 2003) and wolves in the Canadian arctic (average 63,058 km^2^ for males and 44,936 km^2^ for females, Walton et al., 2001). As a result, the lynx in our study site represent a very extreme case of space use relative to body weight among mammalian carnivores.

The explanation for these massive home ranges probably lies in the low environmental productivity and high degree of seasonality of this northern study area (Duncan et al., 2015). This is also exacerbated by the fact that reindeer migrate between seasonal ranges and sheep are brought inside during winter resulting in a major seasonal differences in prey availability (Henden et al., 2014; Walton et al. 2017). As a result, the lynx had access to an abundant supply of prey during summer, whereas in winter some of the lynx had almost no access to reindeer apart from a highly variable number of scattered individuals that remained on summer ranges because they were not gathered during the autumn round‐ups (Walton et al., 2017). Therefore, lynx most likely have to maintain very large annual ranges to allow them to cope with these seasonal fluctuations. In areas and seasons without access to reindeer, the lynx had to prey on a wide diversity of low density prey, including birds, mountain hares, red foxes, and occasionally carrion (Mattisson, Odden, et al., 2011). It should be noted that lynx in our study system did not show any tendency to migrate after the reindeer (Walton et al., 2017), as has also been shown in the similar Sarek ecosystem (Danell et al., 2006).

Another factor that can explain the exceptionally large home range sizes of lynx in this area is the very low proportion of forest available in the study area (lynx are almost exclusively associated with forests in the European part of their range). The majority of the area within the home ranges was open tundra, with forest only constituting around 20%–26% of home range sizes on average (Table 1). No lynx had more than 35% forest, and some had as low as 7% forest. Accordingly the area of forest to which each lynx had access to (Females = 293 km^2^ ± 37, Males = 554 km^2^ ± 84) corresponds much more closely to the average home range size of Scandinavian lynx in more southerly boreal forest habitats (Aronsson et al., 2016; Linnell et al., 2001). However, lynx were not confined to forests in our study area, as they frequently crossed tundra areas and about half of their killed reindeer were in tundra habitats (Mattisson et al., 2015). In all other areas where Eurasian lynx have been studied in western Europe, they are heavily associated with forest habitats. Our study shows that they are able to persist in areas with very low amounts of forest (minimum 60 km^2^ of forested area in lynx range) and that they can utilize open alpine‐tundra habitats when needed. This provides valuable insights into how lynx may live in other parts of their distribution, such as Central Asian grasslands or the Himalayas, where they remain unstudied (Breitenmoser et al., 2015). The results contribute to a growing literature concerning the unique ecologies of populations of species living at the periphery of their distributions (e.g., Koprowski et al., 2008).

A final issue concerns the fact that this population is subject to relatively high rates of human induced mortality (like for all Scandinavian lynx populations, Andrén et al., 2006) and persist at very low densities. Hunting is used to intentionally maintain the population close to its present density to limit conflicts, which minimizes intra‐specific competition for space. The implication is that although resource availability may require lynx to use large areas to satisfy their needs for prey, the management regime may facilitate this by reducing intraspecific competition and allowing animals to move with fewer social constraints (Aronsson et al., 2016; Pesenti & Zimmermann, 2013).

**FIGURE 4:**
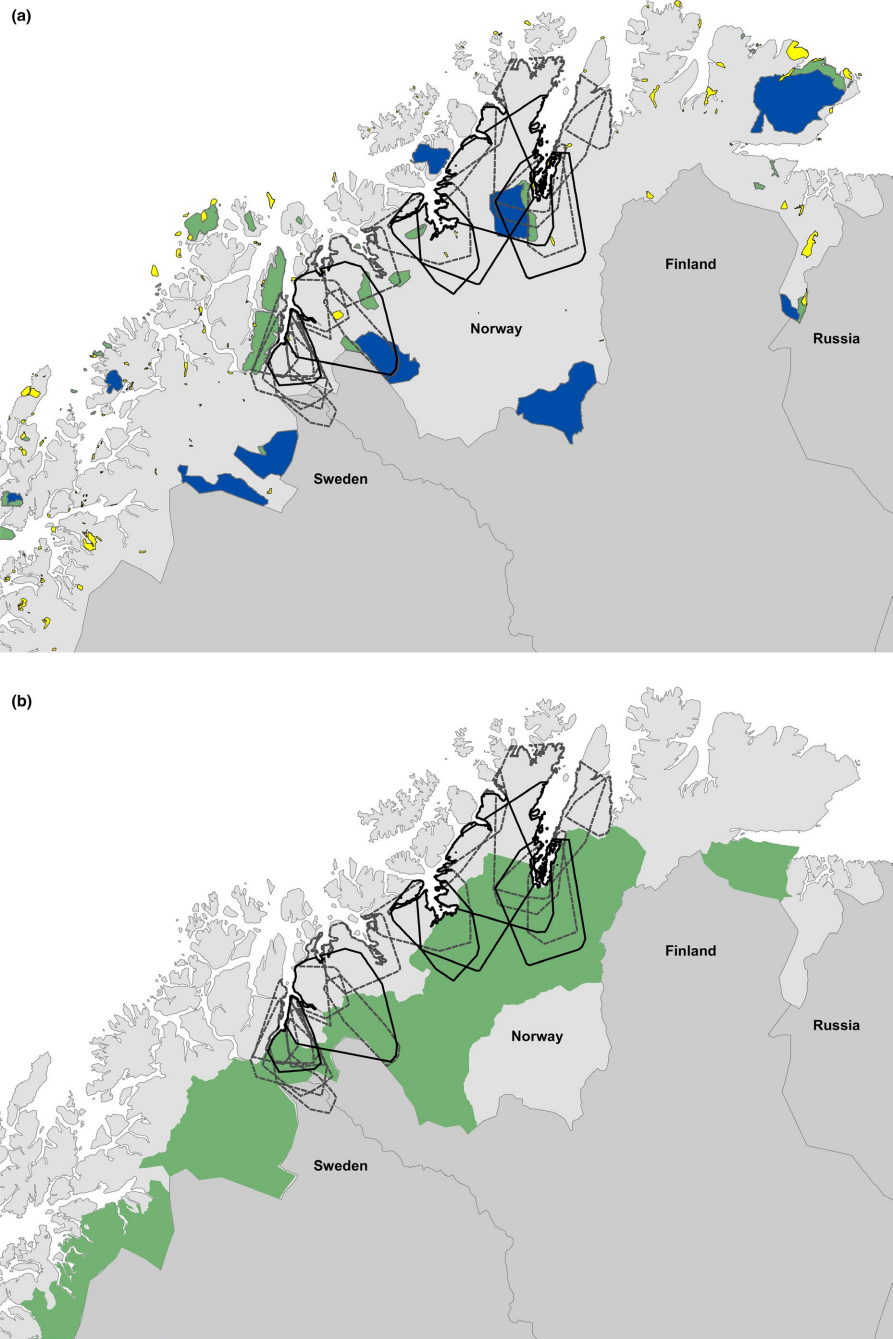
Overlays of annual home range (100% MCP) of Eurasian lynx (*Lynx lynx*) with a) Protected areas, and b) Lynx management zones. In (a), blue areas are national parks, green areas are landscape protection areas, and yellow are nature reserves. In (b), the green areas are lynx management zones, to which lynx reproduction is meant to be confined. The black polygons are individual lynx home ranges, adult males with solid lines, females with dotted lines

With respect to management consequences, these unprecedented home range sizes reveal the inadequacy of existing management zones and low relevance of protected areas. The overlay of lynx home ranges and protected areas shows that no individual lynx home range fitted within a protected area (Figure 4a). Furthermore, all protected areas are also subject to reindeer herding, such that the conflict potential is just as high inside as outside protected areas. This implies that lynx conservation in this region depends 100% on a land‐sharing strategy that mainly occurs outside protected areas, where their main prey are domestic species (reindeer and sheep). This results in the constant need for population regulation to keep the lynx population at a lower level than which it could reach, and the payment of large amounts of damage compensation. The existence of a lynx management zone (Figure 4b) was an attempt to adopt a large scale zoning. However, as the figure shows, the zone is too narrow and is located in the wrong areas from a lynx perspective. The lynx show a strong preference for settling on the coast, which is where the reindeer have their calving areas, and the zone was intended to keep them out of these high conflict areas. However, visual examination of the figures clearly shows how this task is impossible because all individuals regularly leave their designated zones. This underlines that there needs to be an ecological basis (size and location) when constructing administrative zones for wildlife management (Linnell et al., 2005).

In summary, our results describe the presence and ecology of Eurasian lynx at the northernmost part of their global distribution. Furthermore, we document the presence of record home range sizes for a large felid living in an extremely northern environment where their main prey is migratory. There are many direct implications for the spatial management of this species in Norway. However, the ability of individual carnivores to move over such massive areas, and therefore to occur at such low densities, has many implications for designing, and interpreting, field surveys that map species presence and quantify density in general. Finally, the results must serve as a cautionary note when seeking to transfer knowledge between study sites.

## CONFLICT OF INTEREST

The authors declare no conflicts of interest.

## AUTHOR CONTRIBUTION

**John D. C. Linnell:** Conceptualization (equal); Funding acquisition (equal); Project administration (equal); Writing‐original draft (lead); Writing‐review & editing (equal). **Jenny Mattisson:** Conceptualization (equal); Data curation (equal); Formal analysis (lead); Visualization (lead); Writing‐original draft (supporting); Writing‐review & editing (equal). **John Odden:** Conceptualization (equal); Data curation (equal); Formal analysis (supporting); Funding acquisition (equal); Project administration (equal); Writing‐original draft (supporting); Writing‐review & editing (equal).

## Supporting information

Table S1

## Data Availability

Data are uploaded to Dryad digital repository https://doi.org/10.5061/dryad.2v6wwpzmp
