# Supplementary material for: Extreme home range sizes among Eurasian lynx at the northern edge of their biogeographic range
Source: Ecol Evol. 2021 Mar 18;11(10):5001–9. doi: 10.1002/ece3.7436 (PMC8131800; doi:10.1002/ece3.7436)
Supplement: Supplementary file 1 — Table S1 [file ECE3-11-5001-s001.pdf]

Supporting information

Linnell, J.D.C., Mattisson, J. Odden, J. (2021) Extreme home range sizes among Eurasian lynx at the northern edge of their biogeographic range. Ecology and Evolution.

Table S1. Records of lynx home range size (km^2) from publications across Europe. Note that methods vary and even if methods are similar they may not be comparable due to different software settings and location frequencies. Some of the reference also partly include the same data in different publications. The estimates are presented as means (with standard error SE or standard deviation SD) or as ranges (min-max) depending on how the data was presented. The table is sorted by female home range size and our present study is highlighted in grey.

Study area	Females	Males	Method	Reference[1]
Northern Norway	1456 (178 SE)	2605 (438 SE)	MCP 100% (GPS)	This study
Northern Norway	1195 (146 SE)	2243 (331 SE)	Concave polygon (GPS)	This study
North Trøndelag, Central Norway	1076 (284 SE)	1610 (454 SE)	MCP 100% (GPS)	Odden *et al.* (2018)
Northern Norway	916 (132 SE)	1857 (336 SE)	MCP 95% (GPS)	This study
South central Norway	930 (388 SD)	1496 (814 SD)	Kernel 95 % (VHF/GPS)	Boyer *et al* (2015)
Hedmark, Central Norway	832 (206 SD)	1456 (918 SD)	MCP 100% (VHF)	Linnell *et al.* (2001)
Sarek, Northern Sweden	777 (102 SE)	1776 (263 SE)	Concave polygon (GPS)	Mattisson *et al.* (2011)
Hedmark, Central Norway	535 (225 SD)	886 (356 SD)	Kernel 95% (VHF)	Linnell *et al.* (2001)
North Trøndelag, Central Norway	610 (85 SD)	1499 (944 SD)	Kernel 95% (VHF)	Linnell *et al.* (2001)
North Trøndelag, Central Norway	561 (102 SE)	1906 (387 SE)	MCP 100% (VHF)	Sunde *et al.* (2000)
North Trøndelag, Central Norway	561 (70 SD)	1515 (1010 SD)	MCP 100% (VHF)	Linnell *et al.* (2001)
South central Scandinavia	483 (35 SE)	1045 (66 SE)	Kernel 90% (VHF/GPS)	Aronsson *et al.* (2016)
Sarek, Northern Sweden	407 (267 SD)	709 (258 SD)	MCP 100% (VHF)	Linnell *et al.* (2001)
Bergslagen, Central Sweden	307	632 (254 SD)	MCP 100% (VHF)	Linnell *et al.* (2001)
Akershus, Southern Norway	350	812	MCP 100% (VHF)	Herfindal *et al.* (2005)
Jura mountains, Switzerland	75-528	145-1744	MCP 100 % (VHF	Breitenmoser-Würsten *et al.*, 2007
Sarek, Northern Sweden	251 (203 SD)	431 (83 SD)	Kernel 95% (VHF)	Linnell *et al.* (2001)
North Trøndelag, Central Norway	235 (36 SE)	1719 (252 SE)	Kernel 95% (VHF)	Sunde *et al.* (2000)
Bohemian–Bavarian forest	187	599	MCP 100% (GPS)	Magg *et al* 2016
Swiss Jura, Switzerland	168 (64 SD)	264 (23 SD)	MCP 100% (VHF)	Breitenmoser *et al.*, 1993
Balkan	165 (n=1)	466	MCP 100% (GPS)	Melovski et al (2020)
Bialoweiza, Poland	152 (37 SD)	235 (52 SD)	Kernel 95% (VHF)	Schmidt et al 1997
Carpathian Mountains, Poland	124-190	164-199	MCP 100% (VHF)	Okarma *et al* 2007
Bialoweiza, Poland	133 (12 SD)	248 (57 SD)	MCP 100% (VHF)	Schmidt et al 1997
Bohemian–Bavarian forest	122	445	MCP 95% (GPS)	Magg *et al* 2016
Jura mountains, Switzerland	62-224	110-328	Kernel 95% (VHF)	Breitenmoser-Würsten *et al.*, 2007
Balkan	119 (n=1)	373	MCP 95% (GPS)	Melovski et al (2020)
Switzerland	115 (64 SD		MCP 95% (VHF)	Molinai-Jobin et al 2007
Balkan	109 (n=1)	400	Kernel 95% (GPS)	Melovski et al (2020)
Bohemian–Bavarian forest	108	382	MCP 90% (GPS)	Magg *et al* 2016
Bergslagen, Central Sweden	97	305 (117 SD)	Kernel 95% (VHF)	Linnell *et al.* (2001)
Switzerland	80 (33 SD)		Kernel 95% (VHF)	Molinai-Jobin et al 2007

[1] This is not a full review over available literature on lynx home ranges.

References

Aronsson, M., M. Low, J. V. Lopez-Bao, J. Persson, J. Odden, J. D. C. Linnell, and H. Andren. 2016. Intensity of space use reveals conditional sex-specific effects of prey and conspecific density on home range size. Ecology and Evolution 6:2957-2967.

Bouyer, Y., V. Gervasi, P. Poncin, R. C. Beudels-Jamar, J. Odden, and J. D. C. Linnell. 2015. Tolerance to anthropogenic disturbance by a large carnivore: the case of Eurasian lynx in south-eastern Norway. Animal Conservation 18:271-278.

Breitenmoser, U., P. Kavczensky, M. Dotterer, C. Breitenmoserwursten, S. Capt, F. Bernhart, and M. Liberek. 1993. Spatial-Organization and Recruitment of Lynx (Lynx-Lynx) in a Re-Introduced Population in the Swiss Jura Mountains. Journal of Zoology 231:449-464.

Breitenmoser-Wursten, C., F. Zimmermann, P. Stahl, J. M. Vandel, A. Molinari-Jobin, P. Molinari, S. Capt, and U. Breitenmoser. 2007. Spatial and social stability of a Eurasian lynx Lynx lynx population: an assessment of 10 years of observation in the Jura Mountains. Wildlife Biology 13:365-380.

Breitenmoser, U., Kavczensky, P., Dötterer, M., Breitenmoser-Würsten, C., Capt, S., Bernhart, F. & Liberek, M. (1993) Spatial organization and recruitment of lynx (Lynx lynx) in a re-introduced population in the Swiss Jura Mountains. Journal of Zoology, 231, 449-464.

Herfindal, I., J. D. C. Linnell, J. Odden, E. B. Nilsen, and R. Andersen. 2005. Prey density, environmental productivity and home-range size in the Eurasian lynx (Lynx lynx). Journal of Zoology 265:63-71.

Linnell, J. D. C., R. Andersen, T. Kvam, H. Andren, O. Liberg, J. Odden, and P. F. Moa. 2001. Home range size and choice of management strategy for lynx in Scandinavia. Environmental Management 27:869-879.

Magg, N., J. Muller, C. Heibl, K. Hacklander, S. Wolfl, M. Wolfl, L. Bufka, J. Cerveny, and M. Heurich. 2016. Habitat availability is not limiting the distribution of the Bohemian-Bavarian lynx Lynx lynx population. Oryx 50:742-752.

Mattisson, J., Persson, J., Andren, H. & Segerstrom, P. (2011) Temporal and spatial interactions between an obligate predator, the Eurasian lynx (Lynx lynx), and a facultative scavenger, the wolverine (Gulo gulo). Canadian Journal of Zoology-Revue Canadienne De Zoologie, 89, 79-89.

Melovski, D., G. Ivanov, A. Stojanov, V. Avukatov, A. Gonuv, A. Pavlov, U. Breitenmoser, M. von Arx, M. Filla, M. Krofel, J. Signer, and N. Balkenhol. 2020. First insight into the spatial and foraging ecology of the critically endangered Balkan lynx (Lynx lynx balcanicus, Buresh 1941). Hystrix-Italian Journal of Mammalogy 31:26-34.

Molinari-Jobin, A., F. Zimmermann, A. Ryser, P. Molinari, H. Haller, C. Breitenmoser-Wursten, S. Capt, R. Eyholzer, and U. Breitenmoser. 2007. Variation in diet, prey selectivity and home-range size of Eurasian lynx Lynx lynx in Switzerland. Wildlife Biology 13:393-405.

Odden, J., Mattisson, J., Langeland, K., Stien, A. Linnell, J.D.C. & Tveraa, T. 2018. Large carnivores and semi-domestic reindeer in central Norway. Final Report. NINA Report 1380. Norwegian Institute for Nature Research.

Okarma, H., S. Sniezko, and W. Smietana. 2007. Home ranges of Eurasian lynx Lynx lynx in the Polish Carpathian Mountains. Wildlife Biology 13:481-487.

Schmidt, K., W. Jedrzejewski, and H. Okarma. 1997. Spatial organization and social relations in the Eurasian lynx population in Bialowieza Primeval Forest, Poland. Acta Theriologica 42:289-312.

Sunde, P., T. Kvam, P. Moa, A. Negard, and K. Overskaug. 2000. Space use by Eurasian lynxes Lynx lynx in central Norway. Acta Theriologica 45:507-524.
